# Membrane-Inlet Mass Spectrometry Enables a Quantitative Understanding of Inorganic Carbon Uptake Flux and Carbon Concentrating Mechanisms in Metabolically Engineered Cyanobacteria

**DOI:** 10.3389/fmicb.2019.01356

**Published:** 2019-06-25

**Authors:** Damien Douchi, Feiyan Liang, Melissa Cano, Wei Xiong, Bo Wang, Pin-Ching Maness, Peter Lindblad, Jianping Yu

**Affiliations:** ^1^ Biosciences Center, National Renewable Energy Laboratory, Golden, CO, United States; ^2^ Microbial Chemistry, Department of Chemistry-Ångström, Uppsala University, Uppsala, Sweden

**Keywords:** MIMS, carbon uptake rate, cyanobacteria, FbaA, carbon fixation

## Abstract

Photosynthesis uses solar energy to drive inorganic carbon (Ci) uptake, fixation, and biomass formation. In cyanobacteria, Ci uptake is assisted by carbon concentrating mechanisms (CCM), and CO_2_ fixation is catalyzed by RubisCO in the Calvin-Benson-Bassham (CBB) cycle. Understanding the regulation that governs CCM and CBB cycle activities in natural and engineered strains requires methods and parameters that quantify these activities. Here, we used membrane-inlet mass spectrometry (MIMS) to simultaneously quantify Ci concentrating and fixation processes in the cyanobacterium *Synechocystis* 6803. By comparing cultures acclimated to ambient air conditions to cultures transitioning to high Ci conditions, we show that acclimation to high Ci involves a concurrent decline of Ci uptake and fixation parameters. By varying light input, we show that both CCM and CBB reactions become energy limited under low light conditions. A strain over-expressing the gene for the CBB cycle enzyme fructose-bisphosphate aldolase showed higher CCM and carbon fixation capabilities, suggesting a regulatory link between CBB metabolites and CCM capacity. While the engineering of an ethanol production pathway had no effect on CCM or carbon fixation parameters, additional fructose-bisphosphate aldolase gene over-expression enhanced both activities while simultaneously increasing ethanol productivity. These observations show that MIMS can be a useful tool to study the extracellular Ci flux and how CBB metabolites regulate Ci uptake and fixation.

## Introduction

Photosynthesis has been responsible for decreasing CO_2_ in the atmosphere from 30–35% to 0.04% over the last 3 billion years or so (Blankenship, 2010) and is the major physicochemical process that generates organic molecules and, as such, supports most of the life on Earth. The main enzyme responsible for this CO_2_ fixation, ribulose-1,5-bisphosphate carboxylase/oxygenase (RubisCO) appeared 2.5 billion years ago and has evolved to adapt to the decreasing CO_2_ concentrations and concurrently increasing O_2_ levels (Whitney et al., 2011). CO_2_ and O_2_ are competitive substrates for RubisCO, leading to either CO_2_ fixation (carboxylation) or photorespiration (oxygenation). As a result, photosynthetic organisms have evolved various strategies to favor carboxylation over oxygenation by increasing carbon availability for RubisCO.

**Figure 1:**
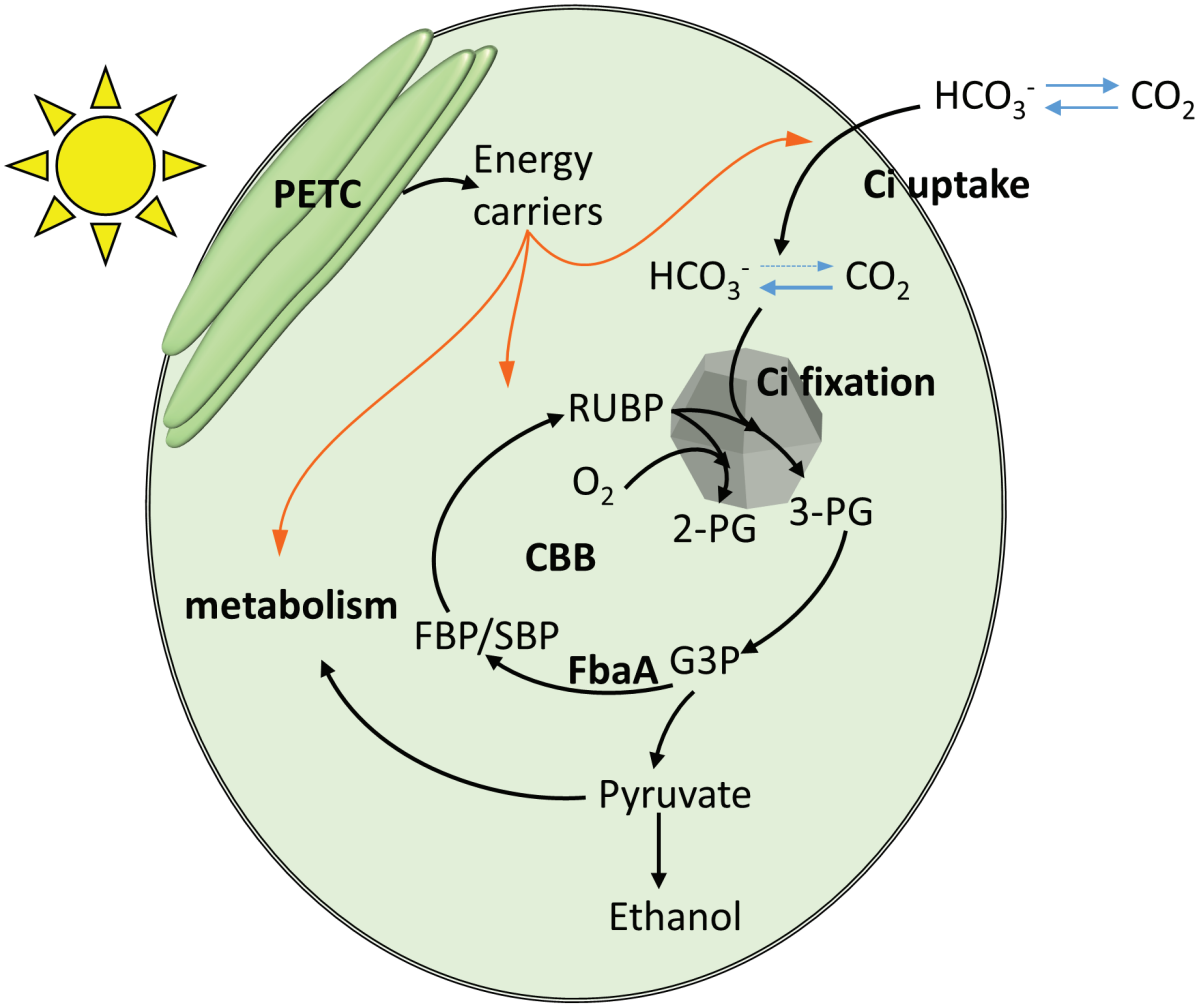
A schematic representation of the relationship between photosynthetically harvested energy by the photosynthetic electron transfer chain (PETC), inorganic carbon (Ci) uptake and fixation, and the anabolic metabolism. Orange arrows indicate chemical energy fluxes. Black arrows indicate carbon flux.

In cyanobacteria, the inorganic carbon (Ci) is concentrated in the cytoplasm to levels over 100 times the external concentration in air-saturated condition (Woodger et al., 2005) *via* carbon concentrating mechanisms (CCM; Figure 1). At least five different uptake proteins or complexes are involved in this process, each with different affinities for Ci. Among them are the BicA and SbtA, sodium/bicarbonate symporters, powered by a sodium gradient across the plasma membrane. NdhD3 and D4 were proposed to be involved in regenerating this sodium gradient, powered by NADPH or Ferredoxin (Wang et al., 2004; Woodger et al., 2007). The involvement of plasma membrane sodium/proton antiporters and ATPase was also hypothesized (Kamennaya et al., 2015). Another bicarbonate transporter is the Bct1 complex, which has its own ATPase activity. In addition, the CO_2_ uptake systems NDH1-3 and NDH1-4 directly convert the CO_2_ to bicarbonate in the cytoplasm using energy from photosynthetic or respiratory thylakoid electron flow (Battchikova et al., 2011; Artier et al., 2018), locking incoming CO_2_ which diffuses freely across the membrane and limiting Ci leakages.

RubisCO is confined to a bacterial micro-compartment, the carboxysome, along with carbonic anhydrase enzymes, which converts HCO_3_^−^ into CO_2_. Together the membrane transporters, cytoplasm and carboxysome carbonic anhydrases form a Ci conduit from the medium to the carboxysome, so that the concentration of CO_2_ within the carboxysome is up to 4,000-fold higher than it is externally (Sültemeyer et al., 1995; Price et al., 1998; Kaplan and Reinhold, 1999; Woodger et al., 2005). RubisCO converts one molecule of ribulose bis-phosphate (RuBP) and one molecule of CO_2_ to two molecules of 3-phosphoglycerate (3PG), which enter the Calvin-Benson-Bassham (CBB) cycle. While RuBP is regenerated, other intermediates are formed and connect to the central carbon metabolism and anabolic pathways, feeding the production of cellular constituents.

An in-depth understanding of photosynthetic mechanism, such as the CCM, requires a more comprehensive, systems-level approach to measure photosynthetic carbon flux within a biochemical network. With respect to photosynthetic organisms, the intracellular carbon flux can be determined through a new ^13^CO_2_/NaH^13^CO_3_ labeling approach: isotopically nonstationary metabolic flux analysis (INST-MFA) (Adebiyi et al., 2015). This method allows the estimation of relative photosynthesis and photorespiration fluxes yielding sugar phosphates, organic acids, and other intracellular metabolites in a model phototroph such as *Synechocystis* sp. PCC 6803 (hereafter *Synechocystis*). To obtain an absolute quantification of fluxomic values, an isotope tracer experiment must be coupled to fundamental determinants of *in vivo* cell physiology. For example, measurements of cell specific rates of nutrient uptake and product formation (i.e., normalized to cell density) allow for intracellular flux calculations using INST-MFA. The measured Ci fixation kinetics is a key input to these methods, because they constrain the solution space of feasible intracellular fluxes. Therefore, an accurate estimation of Ci fixation kinetics and their associated uncertainties is an essential task in the construction of accurate metabolic flux maps for phototrophs.

*In vivo* measurement of Ci utilization rates in a photosynthetic system is challenging. In aqueous solution, dissolved CO_2_ exists in equilibrium with bicarbonate ions, and both forms can be taken up into photosynthetic cells. Ci uptake can be measured by various methods. We have previously used sealed tubes and gas chromatography to measure the difference of Ci concentration over time (Xiong et al., 2015). This method provides the averaged Ci uptake rate over a longer time (hours) but cannot distinguish Ci uptake from fixation kinetics nor measure real time performance at specific Ci concentrations. The isotope labeling method has also been widely used. However, it has a higher leakage rate and suffers from interference caused by non-labeled Ci brought into the growth medium by some of its constituents (Eichner et al., 2015). An alternative technique is to measure gas phase CO_2_ by infrared absorption (Oakley et al., 2012). This technique requires higher cell density, which causes self-shading that affects the reliability of experiments. It also requires large culture volumes and is dependent on the equilibrium of CO_2_ between the gas phase and the liquid phase, which can differ under various conditions including temperature, pressure, medium composition, and pH. Additionally, water vapor has a high absorption coefficient in the infrared region, making the efficiency of the desiccant or compensation mechanisms critical for reliable measurements, especially at very low Ci concentrations. The measurement of O_2_ evolution at different Ci concentrations is also a common method to determine the overall affinity of cells for Ci (Woodger et al., 2005). However, this is an indirect measurement and is complicated by the existence of multiple O_2_ consumption pathways in cyanobacteria and other phototrophs.

In the 1960s, a technique was developed based on the permeation of gases through a silicone membrane which separate the culture media from the high vacuum line that leads to the detector of a mass spectrometer (Hoch and Kok, 1963). This experimental setup, now called MIMS (Membrane-Inlet Mass Spectrometry), was used to measure algal Ci consumption directly and in real time in the growth medium (Radmer and Kok, 1976). This instrument measures only small molecules that can pass through the silicone membrane. While CO_2_ is detectable, carbonates and bicarbonates are not permeable. Therefore, the measure of total inorganic carbon relies on the existing equilibrium that results from the fast interconversion of ${CO}_{2}↔{HCO}_{3}↔{CO}_{3}^{2–}$, which is highly sensitive to the medium pH.

While MIMS was used to study cyanobacterial CCM in the past (Espie et al., 1988; Miller et al., 1988; Tchernov et al., 2003), it has not been widely used in recent years. We adapted this method to measure Ci uptake flux in engineered cyanobacteria quantitatively by determining the extracellular Ci consumption rate per Ci concentration in real time. The kinetic parameters at various Ci concentrations are dependent on intracellular physiology and used to differentiate Ci uptake by CCM and Ci consumption by the carbon fixation machinery. We found that CBB engineering influences the regulation of the Ci uptake as well as fixation activities. We compared Ci uptake flux in wild-type (WT) *Synechocystis* and engineered strains over-expressing fructose-bisphosphate aldolase gene (*fbaA*) either in a WT background or in an ethanol-producing strain (Liang and Lindblad, 2016, 2017). *fbaA* over-expression affects the carbon metabolites in CBB cycle and likely Ci fluxes. Interestingly, the results indicate a positive relationship between the activity of the CBB cycle and the kinetics of CCM, which will help inform genetic engineering strategies of cyanobacterial central carbon metabolism for enhanced carbon utilization.

## Materials and Methods

### Growth of Cyanobacteria

The *Synechocystis* strains used in this study were previously reported (Liang and Lindblad, 2016, 2017). We compared here the WT strain (empty vector control strain with kanamycin resistance, named Km in the below Liang et al. papers) to over-expressing strains generated *via* the same vector. They were grown on a modified BG-11, without added carbonate or bicarbonate, and supplemented with 50 mM NaCl and 20 mM TES, 50 mg/L kanamycin, and filter sterilized. The pH was adjusted to 7.4. Plates were kept under 5% CO_2_, 30°C, 50 μE m^−2^ s^−1^ provided by cool white fluorescent light tubes. The physiological tests were carried out after at least 48 h of incubation in 100 ml of BG-11 in 250 ml baffled Erlenmeyer flasks, continuously air bubbled at 100 ml min^−1^, 30°C, 100 μE m^−2^ s^−1^ of white LED light (4,500 K). The OD_730_ was always kept under 1, and the culture was diluted about 16 h prior to the experiment and harvested at an OD_730_ of about 0.4.

The MIMS set up, operation, and data processing are detailed in the section “Results.”

### Doubling Time

Cultures with an OD_730_ < 1 pre-acclimated for at least 48 h at 100 μE m^−2^ s^−1^ with air bubbling were diluted to an OD_730_ of about 0.050 in pre-warmed 30°C BG-11. The first density measurement was taken 1 h after the dilution. A second measurement was carried out after 16 h, when the OD_730_ was still under 0.5. These two measurements were used to calculate the doubling time.

### Net O_2_ Production

Cultures grown overnight with OD_730_ lower than 0.4 were spun down and resuspended in fresh BG-11 medium to OD_730_ of 0.7–0.8. Samples were incubated in 50 ml falcon tubes in the dark under agitation for 30 min. About 9 ml aliquots of the sample were transferred to the measurement cuvette in the dark and were stirred at 700 rpm and air bubbled for 10 min with 0.0022% antifoam 204, kept at 30°C by water bath. Measurement by MIMS started with this 10-min dark incubation. The air bubbling lead to an air saturated sample and used to calibrate at 278 μM O_2_. When the incubation was completed, the bubbling was turned off, and after 10–20 s, the light was turned to 150 μE m^−2^ s^−1^ (LED, 6,000 K) for 4 min to measure oxygen production. The measurement was maintained for 4 more minutes in the dark to measure respiration. Finally, the sample was bubbled with N_2_ to a steady state to calibrate at 0 μM O_2_.

### High Ci Acclimation

WT strain was cultured in BG-11 medium (with 50 mM NaHCO_3_ replacing the NaCl) for at least 3 days prior the measurement (re-diluted every 24 h), without air bubbling, but under the same stirring, temperature, and light conditions. The cells were harvested by centrifugation with the same procedure described in the Ci uptake method, except that the cells went through an extra step of washing with a medium without HCO_3_^−^.

### Chlorophyll Content Measurement

WT strain and the *fbaA* OE strain were grown overnight from a healthy low Ci-acclimated culture. About 14 ml of culture with OD_730_ < 0.4 were spun down at 2,500 g for 5 min at room temperature, and the pellet was resuspended in 2 ml of −80°C methanol and left overnight at −80°C. After another spinning, the absorbance of the supernatant was measured at 665 and 720 nm. The chlorophyll content was calculated using an equation of μg Chla/ml = 12.9447* (OD_665_-OD_720_) (Ritchie, 2006).

## Results

### MIMS Experimental Setup and the Biological Significance of the Measurement

**Figure 2:**
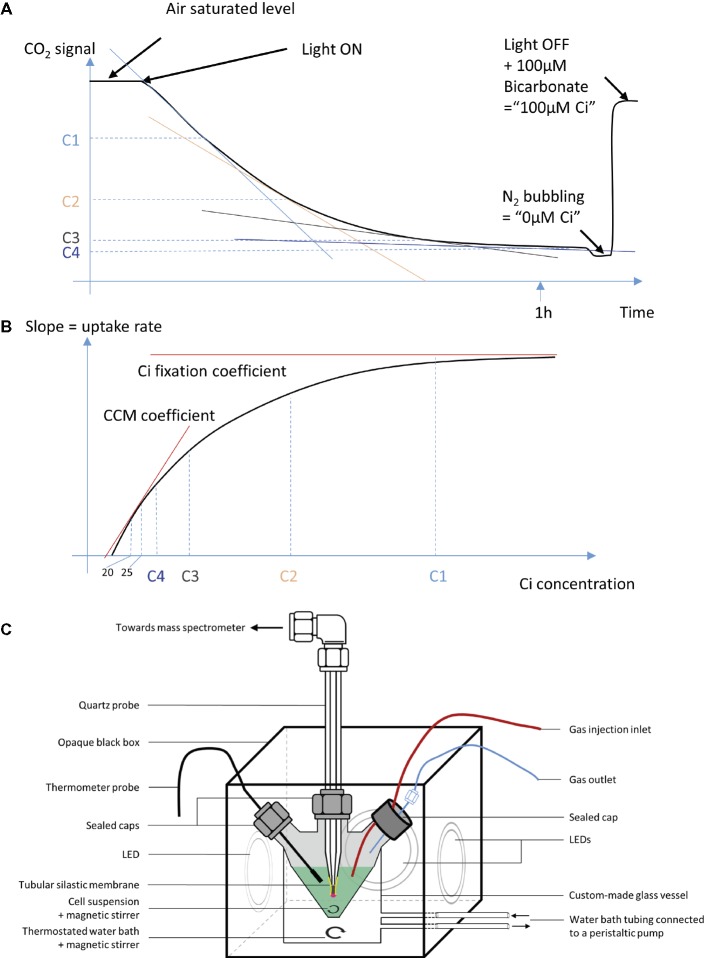
A schematic representation of the MIMS set up and data processing. **(A)** A raw curve typically obtained on the MIMS. C1, C2, C3, and C4 are arbitrary Ci concentrations used to illustrate data processing. **(B)** A plot of the slopes calculated from curve A, over the Ci concentration, as displayed for all Ci uptake assays in this paper. **(C)** The MIMS experimental setup used in this study.

It is widely accepted that CO_2_ diffuses through biological membranes by using aquaporins and a number of other ways (Tchernov et al., 2001). Under high Ci levels at pH 7–8, bicarbonate transporters and other constituents of the CCM are bypassed by the saturating flow of CO_2_ entering the cell. In this work, Ci uptake was studied under conditions where all CCM components are actively involved. Air bubbling into the cultures was used to maintain a low but constant Ci supply while avoiding over accumulation of the O_2_ generated by the photosynthetic electron transport chain (PETC). Cultures were maintained in BG-11 medium below an OD_730_ of 0.4, where self-shading is minimal, and nutrients are replete. Cells were harvested by centrifugation and re-suspended in fresh BG-11 media to an OD_730_ of 0.5. For consistent results in this measurement, the same media batch was used for all compared samples and replicas. About 9 ml aliquots of this suspension were subjected to CO_2_ concentration monitoring by MIMS (HIDEN HAS-301-1503A) using the SEM detector (settle time 400 ms, dwell time 2,750 ms). The sample was injected into a 20 ml measurement cuvette, which was custom designed and crafted (Allen Glass, Boulder, Colorado; Figure 2). In such conditions, buildup of photosynthetic oxygen was minor, which was confirmed by MIMS measurements. The pH was observed to be constant during the course of the experiment due to the presence of 20 mM TES in BG11 media, compared to the 100 μM of Ci consumed. The suspension was completed with 0.0022% antifoam 204 (Sigma), stirred at 700 rpm, and bubbled with air for 8 min 30 s in 10 μE m^−2^ s^−1^ (room light). The MS measurement was then commenced, and the bubbling stopped (T0). The measurement vessel was sealed, and the light was turned on to about 200 μE m^−2^ s^−1^ (white LED at 6,000 K). Dissolved CO_2_ was measured over time, from T0 when the sample is air saturated, to T-final when CO_2_ consumption becomes undetectable. A calibration was performed after each sample, where zero Ci was set by bubbling the sample with N_2_ for 10 min, and 100 μM Ci was set by an injection of freshly prepared NaHCO_3_ at pH 7.4. The concentration of dissolved CO_2_ was considered a readout of the total Ci.

While it was hypothesized that in some cases, at the RubisCO vicinity, the consumption of CO_2_ can drive an imbalance in the CO_2_ HCO_3_^−^ equilibrium (Sun et al., 2019), it is compensated intracellularly by carbonic anhydrases. The measurement of extracellular CO_2_ could also possibly be interfered by an imbalanced Ci hydration equilibrium. However, in our conditions, it was shown that *Synechocystis* uptakes more HCO_3_^−^ than CO_2_ (Benschop et al., 2003), yet we observed an immediate decrease in CO_2_ readings upon illumination as illustrated in Figure 2A (data not shown), indicating equilibrium of the Ci species. This is also supported by the absence of growth delay in cytoplasmic carbonic anhydrase mutant, indicating that the natural homeostasis between the two Ci species is able to support growth in laboratory conditions (So et al., 1998). In addition, the conditions we use are moderate in terms of pH, light, and cell density, thus unlikely to drive extracellular Ci out of equilibrium. Based on (Dreybrodt et al., 1996) and our calculation, at the time scale of 1 h experiment, we consider that the CO_2_ hydration is not limiting.

Because *Synechocystis* acclimation to low Ci concentration begins after about 2 h (Benschop et al., 2003), our method was designed so that each individual measurement did not last more than an hour.

The Ci uptake rate was calculated from the slope of the Ci concentration versus time over a 20 timepoint window, and this rate was plotted against the Ci concentration of the first point used to calculate the slope. A fitting curve was calculated using a five-parameter Hill modified equation.

$$Y=Y0+\frac{a{\left(X–X0\right)}^{b}}{c^{b}+{\left(X–X0\right)}^{b}}$$

*Y* is the slope, plotted in ordinate; *X* is the Ci concentration plotted in abscissa; *a, b, c, Y*0, and *X*0 are variables used to fit the curve to experimental data.

**Figure 3:**
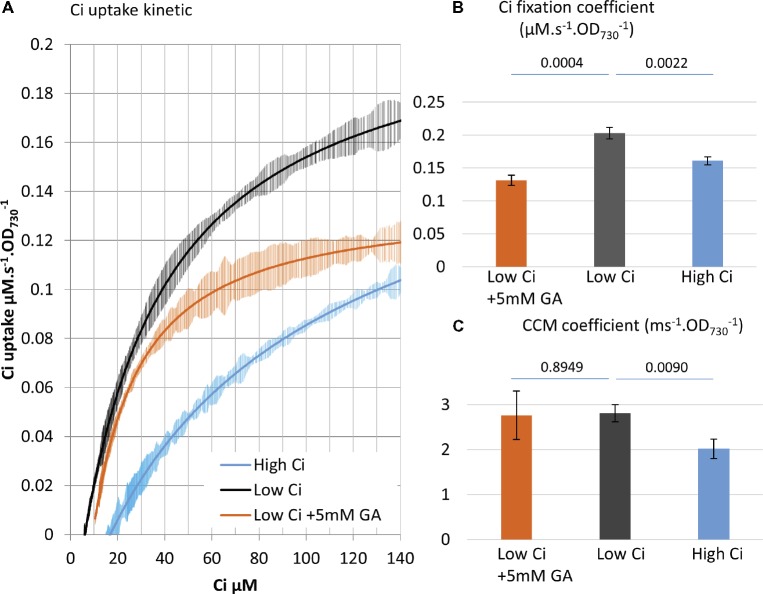
MIMS can delineate Ci fixation reactions versus CCM operation. **(A)** Ci consumption rate over media Ci concentration is shown for the low Ci (air bubbling) acclimated WT strain in black. The effect of the CBB cycle inhibitor glycolaldehyde (GA) at sub-saturating concentration (5 mM) on the kinetic is shown in orange. Data from 48 h high Ci (50 mM sodium bicarbonate) acclimated WT cultures are shown in blue. All curves display 20 point standard deviations as vertical bars. (**B).** The carbon fixation coefficient and **(C)** the CCM coefficient calculated from the curves in **(A)**. *p* from one-sided comparison student *t*-test are shown, *n* = 3.

As shown in Figures 2, 3, the MIMS method provides real-time Ci uptake rate over a range of external Ci concentrations. At higher external Ci concentrations (air bubbling), Ci import into cells is considered not limiting, and the measured Ci uptake rates are limited by carbon fixation reactions. In contrast, as external Ci concentrations drop toward zero, CCM activity should become the rate limiting step for the measured Ci uptake rates. Thus, the fitting curve can provide quantitative measurement of both CCM activity and Ci fixation (CBB cycle) activity. On the low Ci end, we set 20 μM Ci as the reliable detection limit; thus, the region between 20 and 25 μM Ci on these fitting curves was used to calculate an initial slope, designated as the *CCM coefficient*. On the high Ci end, we designate the *Ci fixation coefficient* as the maximum of the fitting sigmoid, representing Ci fixation rate at Ci sufficient conditions. The kinetic observed here is non-Michaelian, common for multicomponent reactions. Thus, the parameters described are not *V*_max_ and *K*_m_. Other parameters may be calculated from the curve as our understanding of Ci uptake advances.

Designation of the two coefficients allows quantitative differentiation between Ci fixation and CCM activities. An inhibitor study was performed to support this differentiation. The partial inhibition of the CBB cycle with 5 mM of glycolaldehyde, which targets the phosphoribulokinase (Miller and Canvin, 1989), does not affect CCM (Rotatore et al., 1992). When this inhibitor was used in the MIMS method, we observed partial inhibition of Ci uptake in the upper phase (−35%) but not the lower phase of the curve as predicted (Figure 3). At the lower phase, Ci becomes very low, and the Ci supply to the Ci fixation reactions *via* CCM activity becomes limiting. The results obtained here indicate that the lower Ci phase of the uptake curve can serve as an indicator of CCM.

We found that the growth phase and cell density of the culture were critical factors for data reproducibility. We also observed that WT strains from different laboratories do not always behave similarly; some do not induce complete CCM when placed in air bubbling.

### Acclimation to High Ci Conditions Involves a Concurrent Decline of the CO_2_ Fixation and CCM Coefficients

The acclimation of *Synechocystis* to high Ci concentrations, from bicarbonate addition or bubbling with CO_2_ enriched air, triggers a decrease in the level of active transporters in CCM (Burnap et al., 2015; Holland et al., 2016), and their apparent affinities for Ci (Skleryk et al., 2002; Benschop et al., 2003). We attempted to verify the previous observation of a delayed acclimation of the CCM machinery to high Ci (Benschop et al., 2003) using the MIMS method and parameters.

**Figure 4:**
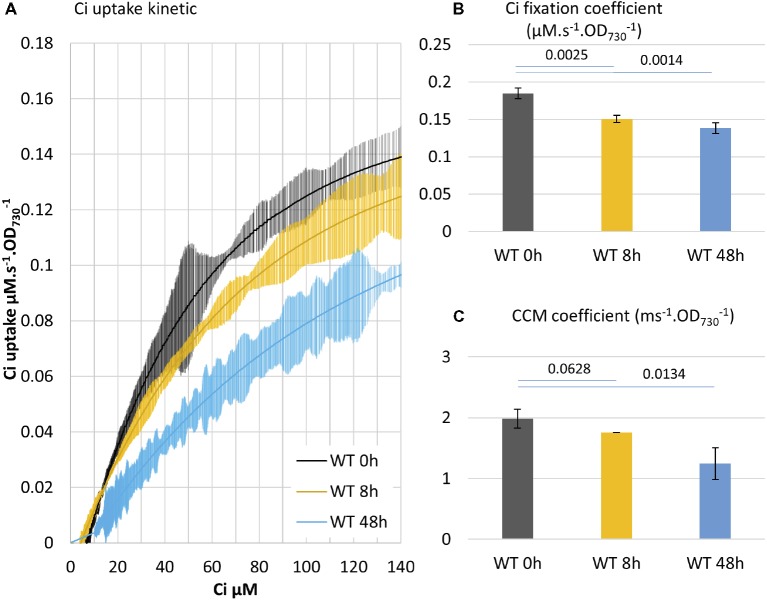
Acclimation to high Ci conditions involves a concurrent decline of Ci fixation and CCM coefficients. **(A)** Delineation of the Ci uptake rate versus Ci concentration. Cultures acclimated for 3 days in air bubbling were supplemented with 25 mM NaHCO_3_ at 0 h and were subjected to the Ci uptake kinetic measurement at 8 h and 48 h. Cells were kept under OD_730_ 0.4 by daily dilution. All curves display 20 points standard deviations as vertical bars. **(B)** The carbon fixation coefficient and **(C)** the CCM coefficient calculated from the curves in **(A)**. *p* from two-tailed comparison student *t*-test are shown, *n* = 3.

A WT culture adapted to low Ci was subjected to the addition of a saturating level of bicarbonate (50 mM) and sampled for MIMS measurement at 0, 8, or 48 h. Acclimation to high Ci conditions affected both the CCM coefficient and Ci fixation coefficient, but with different timing: the Ci fixation coefficient decreased by 19% in 8 h, while the CCM coefficient just started to decrease (note higher *p*). This is followed by a 25% decrease in the Ci fixation coefficient in 48 h, when the CCM coefficient decreased by 37% (Figure 4). These observations are in agreement with prior results (Benschop et al., 2003) and suggest that synthesis/activity of CCM and CBB enzymes are both regulated during adaptation to high Ci conditions. The regulation interestingly appears to be shifted in time. They also further support the assignation of the two phases of the curve to CCM and carbon fixation, respectively.

### Energy Input Affects Both CCM and Ci Fixation as Exhibited by MIMS Measurement

**Figure 5:**
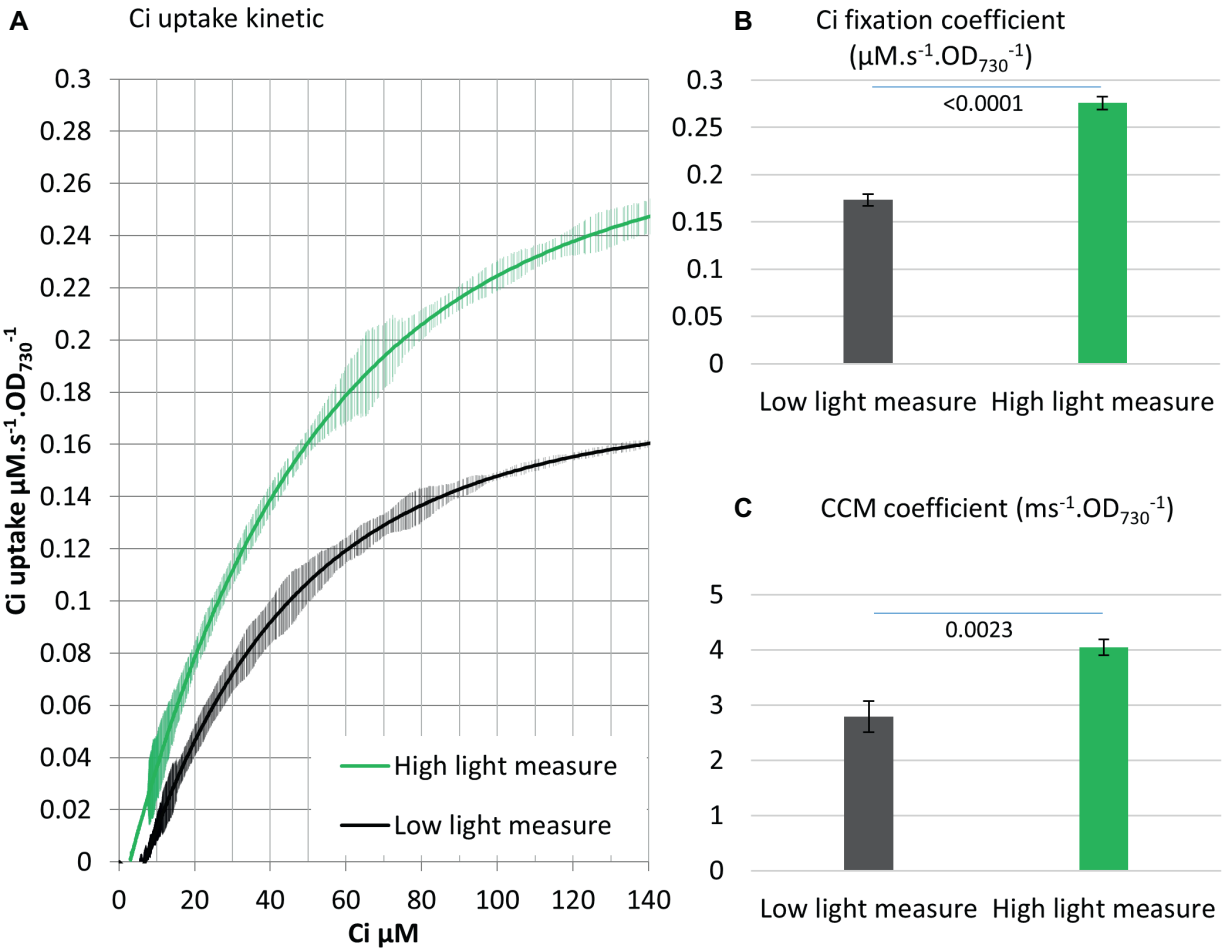
Energy input affects both CCM and Ci fixation. **(A)** Ci consumption rate over media Ci concentration is shown for the low Ci acclimated WT strain. Ci uptake was either measured in 200 μE m^−2^ s^−1^ light as in all other experiments (black) or 750 μE m^−2^ s^−1^ light (green). All curves display 20 points standard deviations as vertical bars. **(B)** The carbon fixation coefficient and **(C)** the CCM coefficient calculated from the curves in **(A)**. *p* from two-tailed comparison student *t*-test are shown, *n* = 3.

Both the concentrating of Ci and the fixation of Ci *via* the CBB cycle requires energy input. The energy needed for those processes is provided by PETC, which harvests light energy and carries out a series of redox reactions leading to the production of energy carriers, mainly NADPH and ATP (Figure 1). We tested the influence of a higher light intensity (200 vs. 750 μE m^−2^ s^−1^) on the Ci uptake kinetics and observed enhanced Ci fixation (+60%) and CCM (+45%) coefficients (Figure 5). While Ci uptake increased with increasing light intensity as previously reported (Kranz et al., 2010), our method further indicates that energy input can be a rate limiting step for both Ci concentrating and Ci fixation activities in *Synechocystis*.

### Over-Expression of the CBB Cycle Enzyme *fbaA* Gene Impacts CCM and Ci Fixation

**Figure 6:**
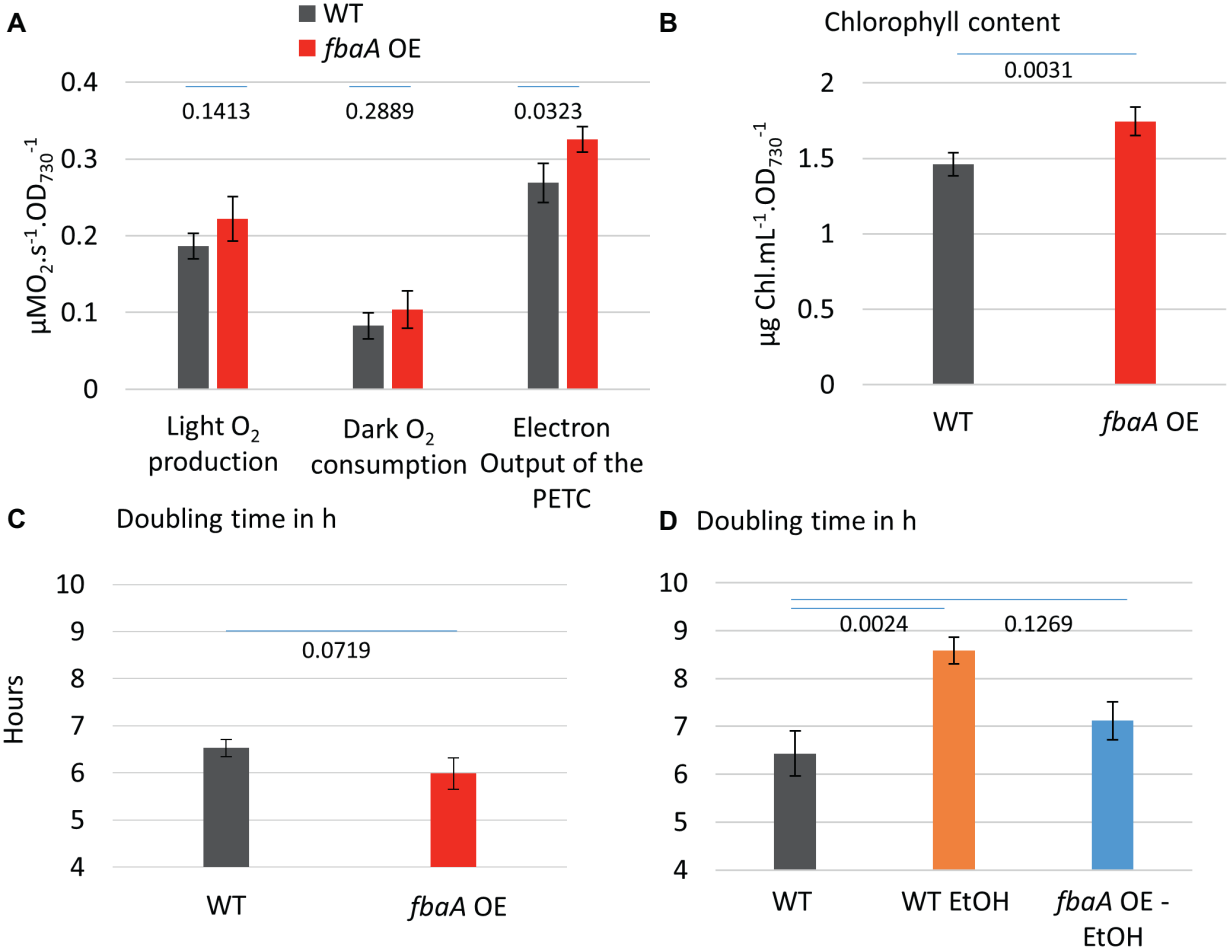
*fbaA* over-expressing strain outperforms WT in multiple aspects **(A)** O_2_ evolution and dark respiration of the WT and *fbaA* OE. Net oxygen evolution rate and dark respiration rate in a closed cuvette are used for the estimation of PETC output at 200 μE m^−2^ s^−1^ light, 30°C, and prior air bubbling. **(B)** Chlorophyll content relative to the OD_730_. **(C)** Doubling time in the growth conditions including 100 μE m^−2^ s^−1^ light, 30°C, and air bubbling. **(D)** Doubling time of ethanol-producing strains with or without simultaneous over-expression of *fbaA. p* from one-sided comparison student *t*-test are shown, *n* = 3.

**Figure 7:**
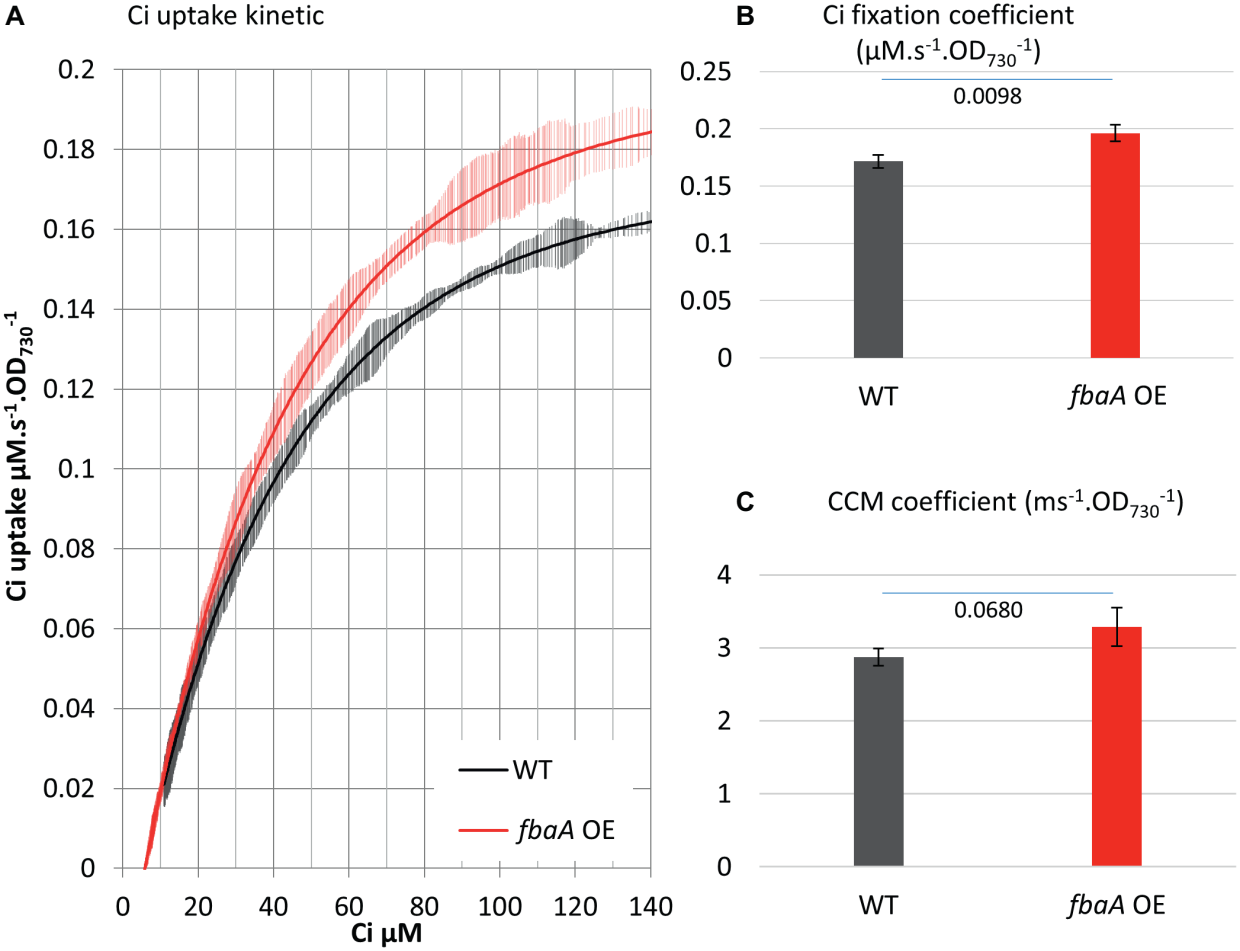
*fbaA* over-expression impacts CCM and Ci fixation. **(A)** Ci consumption rate over media Ci concentration is shown for the low Ci acclimated WT (black) or *fbaA* OE (red). All curves display 20 points standard deviations as vertical bars. **(B)** The carbon fixation coefficient and **(C)** the CCM coefficient calculated from the curves in **(A)**. *p* from one-sided comparison student *t*-test are shown, *n* = 4.

Central carbon metabolites, such as 2-phosphoglycerate (2PG), RuBP, 3PG, and alpha-ketoglutarate (AKG), as well as the electron carrier NAD(P)^+^ and the size of the intracellular Ci pool play an important role in the transcriptional regulation of cyanobacterial Ci uptake activity (Burnap et al., 2015). Based on these observations, we postulated that substrate regeneration by the CBB cycle could be engineered to enhance Ci uptake and fixation. We tested this hypothesis using engineered strains, including an ethanol-producer, and strains over-expressing the fructose bisphosphate aldolase gene (*fbaA OE*) alone or in combination with the ethanol-producer (Liang and Lindblad, 2016; Liang et al., 2018). MIMS was used to evaluate the effects of FbaA on carbon uptake and fixation machinery and to further characterize the effects of *fbaA OE* in carbon accumulation. FbaA is involved in two reactions within the CBB cycle. First, it catalyzes the combination of erythrose-4-phosphate (E4P) and dihydroxyacetone phosphate (DHAP) to form sedoheptulose-1,7-bisphosphate (SBP). Second, it also combines glyceraldehyde-3-phosphate (G3P) with DHAP to form fructose-1,6-bisphosphate (FBP). The f*baA OE* strain grew faster (6.0 vs. 6.5 h^−1^), had a higher chlorophyll content (+20%), and had a higher net O_2_ evolution rate (+21%) compared to the WT (Figure 6). Both the Ci fixation and the CCM coefficients were about 14% higher in *fbaA OE* than WT (Figure 7).

### *fbaA* Over-Expression Enhances Ci Uptake and Fixation in an Ethanol-Producing Strain

**Figure 8:**
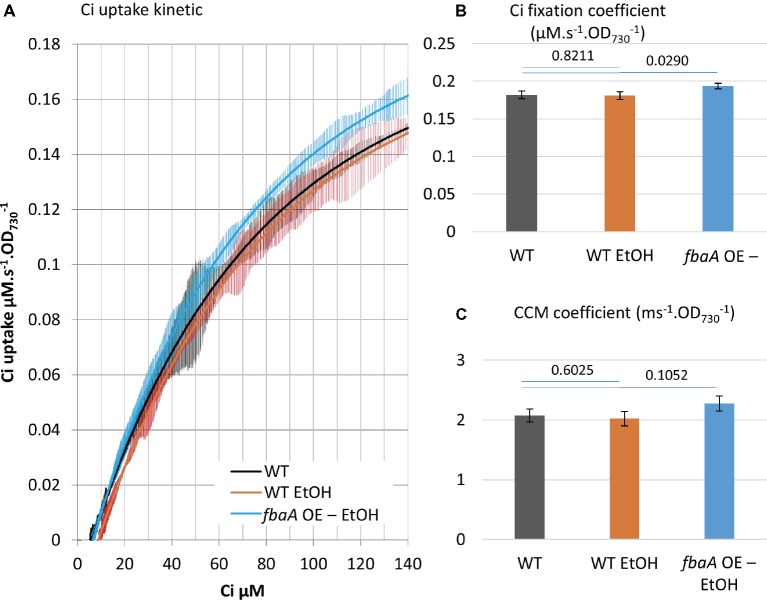
*fbaA* over-expression enhances Ci uptake and fixation in an ethanol-producing strain. **(A)** Ci consumption rate over media Ci concentration is shown for the low Ci acclimated WT (black) and the ethanol-producing strain in WT background (WT EtOH, orange) or a *fbaA* OE background (*fbaA* OE – EtOH, blue). All curves display 20 points standard deviations as vertical bars. **(B)** The carbon fixation coefficient and **(C)** the CCM coefficient calculated from the curves in **(A)**. *p* from one-sided comparison student *t*-test are shown, *n* = 3.

Many cyanobacterial strain engineering efforts involve the production of a target metabolite that is volatile or excreted into the medium, thus expanding the cell’s metabolic sinks. A number of such strains are reported to show enhanced carbon fixation although there is limited knowledge at the molecular level on how this enhancement is achieved (Nozzi and Atsumi, 2015; Xiong et al., 2015; Gao et al., 2016; Zhou et al., 2016). As a test case, we measured Ci uptake parameters in an ethanol-producing strain over-expressing pyruvate decarboxylase and alcohol dehydrogenase from *Zymomonas mobilis* (Luan et al., 2015; Liang and Lindblad, 2016, 2017; Liang et al., 2018). Over-expression of these two ethanol pathway genes and ethanol production had no effect on Ci uptake and fixation kinetics compared to WT (Figure 8). The additional *fbaA OE* feature in this strain nearly doubled ethanol productivity (Liang et al., 2018). We compared the growth rates of the WT and the two ethanol-producing strains. The introduction of the ethanol pathway increased the doubling time (8.6 h^−1^) versus WT (6.4 h^−1^), while the *fbaA* over-expression in the ethanol-producer restored WT like growth (7.1 h^−1^) (Figure 6D). The doubling time of the *fbaA* OE in a WT background, on the other hand, was about 10% shorter than the WT (Figure 6C). While expressing ethanol production genes alone had no effect on Ci uptake and fixation kinetics, their co-expression with *fbaA OE* enhanced both CCM and Ci fixation parameters (10 and 7%, respectively; Figure 8), comparable to the enhancement observed in WT background (Figure 6).

## Discussion

### The MIMS Method Can Quantify Both Ci Concentrating and Fixation Activities

Photosynthesis is nature’s primary Ci utilization pathway and is the basis for developing phototrophs biotechnology for enhanced Ci utilization and the production of fuels and chemicals. Toward this end, a standard and simple method is needed to measure the Ci concentrating and fixating activities *in vivo*. Accurate measurement of Ci uptake rate is also essential for metabolic flux analysis. In this study, a MIMS method that is capable to measure Ci uptake in real time in declining external Ci concentrations is used to quantify CCM and Ci fixation kinetics separately. The results from three different conditions – inhibitor of the CBB, acclimation to high Ci, and increase of the light intensity during the measurement – were in agreement with literature and further support the dual phase readings we report here (Rotatore et al., 1992; Benschop et al., 2003; Kranz et al., 2010; Burnap et al., 2015; Holland et al., 2016).

The MIMS data yielded Ci uptake rates that were then fitted to a plot against the Ci concentration using the Hill equation. The fit allowed calculation of the Ci fixation coefficient reflecting the organism’s maximum Ci uptake rate in air bubbling conditions, as well as the CCM coefficient reflecting the organism’s carbon concentrating capability. The distinction between these two parameters was confirmed by experiments using a sub-inhibitory concentration of the CBB cycle inhibitor glycolaldehyde. Future studies may assign specific CCM mechanisms to various regions along the curve based on Ci affinity, enabling more detailed studies of the individual mechanisms. The MIMS method can also potentially accommodate variations in light intensity and other physiological parameters during measurement.

### Genetic Modification of the CBB Cycle Can Stimulate Both Ci Fixation and Uptake Reactions

Genetic modification of cyanobacterial central carbon metabolism can positively impact culture growth and carbon fixation. However, there has been limited understanding of how the enhancement of carbon utilization is achieved. Here, we tested Ci uptake parameters in a strain over-expressing the *fbaA* gene and in ethanol-producing strains with and without concomitant over-expression of the *fbaA* gene. While it is known that metabolites from the central carbon metabolism directly regulate the expression of CCM genes (Burnap et al., 2015; Orf et al., 2016), it has not been shown *in vivo* whether modifying the metabolic flux in CBB cycle impacts CCM activity. Our observation that *fbaA OE* increased both the Ci fixation coefficient and the CCM coefficient (Figure 7) provides *in vivo* evidence of a regulatory link between CBB metabolites and CCM enhancement. The *fbaA OE* strain is expected to have a modified metabolic flux toward the regeneration of RuBP and the depletion of 3PG. Our data indicate that the enhancement of RuBP regeneration in the CBB cycle likely improves the overall Ci concentrating and fixation processes. The increased fluxes of Ci uptake and CBB cycle demand more energy, which is provided by an increased photosynthetic machinery activity and light harvesting. This was evidenced by about 20% increase in O_2_ evolution rate and chlorophyll content (Figures 6A,B). The stimulation of photosynthesis, including CCM, by CBB cycle modification represents another example of metabolic plasticity in cyanobacteria (Xiong et al., 2017).

The mechanism by which *fbaA OE* regulates CBB activity has been studied in transgenic plants. It was observed that FbaA activity affected photosynthetic efficiency, and its activity can be linked to the CBB metabolic balance and carbon partitioning in potatoes (Haake et al., 1998). Changes in CBB metabolite flux or concentrations are thought to trigger CBB enzymes regulation, likely through gene expression (Henkes et al., 2001; Cai et al., 2016; Simkin et al., 2017). Although contradictory results were found in other plants (Uematsu et al., 2012), all studies agree that the enhancement of photosynthetic activity is likely limited by 3PG depletion and RuBP regeneration. The enhancement of the overall carbon fixation by *fbaA OE* is even stronger at higher Ci conditions (Uematsu et al., 2012), where the rate of Ci fixation by RubisCO depends on the ability of the CBB cycle to regenerate RuBP (Raines, 2003; Ma et al., 2005; Farazdaghi, 2011), assuming that RubisCO quantity itself is not limiting under high Ci conditions (Kanno et al., 2017). Whether the increase in the Ci fixation coefficient in *fbaA OE* cyanobacterium is due to CBB metabolite levels, changes and/or enzyme rearrangement remain to be studied.

In contrast, the mechanism by which *fbaA OE* regulates CCM may be suggested from prior studies of cyanobacterial CCM gene expression in response to metabolite signaling (Burnap et al., 2015). Metabolites, including 2PG, RuBP, 3PG, and AKG, as well as cofactor NAD(P) directly affect major LysR-type transcriptional regulators such as the NAD(P)H dehydrogenase regulator (NdhR), the *cmp* operon regulator (CmpR), and the *rubisCO* operon regulator (RbcR) (Burnap et al., 2015). The internal concentration of Ci itself and the Ci/O_2_ ratio may also be a major signal for gene regulation (Woodger et al., 2005). Further studies of our strains, including quantification of CBB metabolites and transcriptome/proteome analyses, will be needed to clarify the regulatory mechanisms involved in photosynthetic enhancement by *fbaA* OE. In general, the positive effects on Ci utilization in some engineered strains could be attributed to metabolites/transcription regulation pathways as described above and may also depend on biochemical constraints, abundance of transporters, affinity, and the half-lives of the proteins involved in the process. The MIMS method can determine *in vivo* how engineering affects RubisCO Ci fixation kinetics and/or carbon concentrating kinetics and thus help delineate these regulatory mechanisms.

Our observations in *fbaA* OE cyanobacterium extend to engineered strains producing ethanol. The production of ethanol alone negatively impacted the growth rate, suggesting that the diversion of pyruvate toward the ethanol pathway depleted carbon flux toward anabolic metabolism. This negative effect was compensated by the simultaneous *fbaA* OE in the combined strain, in which ethanol production doubled and the growth rate was restored close to WT levels, while both CCM and Ci fixation coefficients increased (Figure 8). It appears that the loss of carbon to ethanol production is compensated in the combined strain by an increase in Ci uptake and metabolic fluxes within the CBB cycle, therefore providing enough 3PG to feed both ethanol production and cell growth. These observations suggest that both growth and ethanol production are limited by Ci uptake and fixation in the ethanol-producing strain, while *fbaA* OE stimulated Ci uptake and fixation, thus improving both ethanol production and growth rate.

As discussed above, *fbaA* OE may lead to a modified balance between metabolites that may enhance the regeneration phase of the CBB cycle and therefore speeds up the RubisCO reactions. A similar hypothesis was made about the over-expression of another CBB enzyme gene, *fsbp*, in the green alga *Chlorella* (Yang et al., 2017). Indeed, it is likely that the increased accumulation of other enzymes favoring the recycling of RuBP would also enhance Ci fixation. The benefit of increased growth potential is then balanced by the cost of producing additional proteins. It could be postulated that the metabolic changes within the CBB cycle would be different by over-expressing one gene versus another. If a step in the regulation of the CCM is modified by intermediate products of the CBB cycle, it is expected that the phenotype would be different among different overexpressers. Preliminary data show that a *Synechocystis* strain over-expressing the fructose/sedoheptulose bisphosphatase gene displays an increase in Ci fixation and a decrease in Ci uptake (data not shown). Thus, over-expression of genes for different central metabolism enzymes could provide more complete information on the regulation of Ci uptake, fixation, and carbon partitioning. Investigating how such changes in the metabolite balance of engineered strains improves or impairs Ci uptake machinery and Ci fixation could guide future strain development for enhanced carbon utilization toward production of fuels and chemicals.

The dynamics of Ci consumption from genetically modified strains and cultures adapted to different growth conditions can be precisely measured using a simple reactor system equipped with MIMS. Such measurements can provide additional information or constraints for model-based ^13^C-metabolic flux analysis and thus contribute to systematic studies of photosynthetic phenotype and fluxome.

## Author Contributions

DD and JY conceived the study and drafted the manuscript. DD conducted the experiments. FL and PL provided engineered strains. FL, MC, BW, WX, P-CM, PL, and JY assisted with experimental design, data interpretation, and troubleshooting. All authors edited and approved the manuscript. We thank Ms. Sunnyjoy Dupuis for language editing.

## Conflict of Interest Statement

The authors declare that the research was conducted in the absence of any commercial or financial relationships that could be construed as a potential conflict of interest.
